# Accelerators: Sparking Innovation and Transdisciplinary Team Science in Disparities Research

**DOI:** 10.3390/ijerph14030225

**Published:** 2017-02-23

**Authors:** Carol R. Horowitz, Khader Shameer, Janice Gabrilove, Ashish Atreja, Peggy Shepard, Crispin N. Goytia, Geoffrey W. Smith, Joel Dudley, Rachel Manning, Nina A. Bickell, Maida P. Galvez

**Affiliations:** 1 Center for Health Equity and Community Engaged Research, Icahn School of Medicine at Mount Sinai, New York, NY 10029, USA; crispin.goytia@mountsinai.org (C.N.G.); nina.bickell@mssm.edu (N.A.B.); maida.galvez@mssm.edu (M.P.G.); 2 Department of Medicine, Icahn School of Medicine at Mount Sinai, New York, NY 10029, USA; janice.gabrilove@mssm.edu (J.G.); ashish.atreja@mssm.edu (A.A.); 3 Department of Genetics and Genomic Sciences, Icahn School of Medicine at Mount Sinai, New York, NY 10029, USA; shameer.khader@mssm.edu (K.S.); joel.dudley@mssm.edu (J.D.); 4 Department of Genetics and Genomic Sciences, Institute of Next Generation Healthcare, Icahn School of Medicine at Mount Sinai, New York, NY 10029, USA; 5 WE ACT for Environmental Justice, New York, NY 10031, USA; peggy@weact.org; 6 Digitalis Ventures, New York, NY 10036, USA; gws16@mac.com; 7 Fordham Law School, New York, NY 10023, USA; rjmanning8@gmail.com; 8 Department of Environmental Medicine and Public Heath, Department of Pediatrics, Icahn School of Medicine at Mount Sinai, New York, NY 10029, USA

**Keywords:** disparities, accelerator, translational research, team science, community engagement, environmental health, genomics, big data, digital health

## Abstract

Development and implementation of effective, sustainable, and scalable interventions that advance equity could be propelled by innovative and inclusive partnerships. Readied catalytic frameworks that foster communication, collaboration, a shared vision, and transformative translational research across scientific and non-scientific divides are needed to foster rapid generation of novel solutions to address and ultimately eliminate disparities. To achieve this, we transformed and expanded a community-academic board into a translational science board with members from public, academic and private sectors. Rooted in team science, diverse board experts formed topic-specific “accelerators”, tasked with collaborating to rapidly generate new ideas, questions, approaches, and projects comprising patients, advocates, clinicians, researchers, funders, public health and industry leaders. We began with four accelerators—digital health, big data, genomics and environmental health—and were rapidly able to respond to funding opportunities, transform new ideas into clinical and community programs, generate new, accessible, actionable data, and more efficiently and effectively conduct research. This innovative model has the power to maximize research quality and efficiency, improve patient care and engagement, optimize data democratization and dissemination among target populations, contribute to policy, and lead to systems changes needed to address the root causes of disparities.

## 1. Introduction

Efforts to move beyond documenting existence and reasons for racial, ethnic and socioeconomic disparities in health and develop effective, sustainable, scalable interventions that advance equity have met with insufficient success. In part, this is because there is inadequate engagement of the stakeholder groups who understand and can impact root causes of disparities, a paucity of focus on diverse populations in research, and a lack of research infrastructure designed to rapidly and efficiently focus teams on emerging equity questions and opportunities [1]. Thus, innovation in translational disparities research is essential.

Research traditionally takes place in disciplinary, disease and demographic silos. Team science has begun to challenge traditional ways of thinking about and conducting scientific endeavors, and highlights the need for researchers and other stakeholders to be educated and attuned to the nuances and best practices for working in teams to spark discovery and inform solutions [2]. Characteristics inherent to fostering a productive investigative team environment and a culture of transdisciplinary research include participatory goal setting, encouraging inclusiveness to foster social cohesiveness, organizing research tasks to be structurally interdependent, encouraging sustained collaboration, and rewarding collaborative processes and achievements through interdependent incentive systems [3,4,5].

While disparities researchers have been pioneers in stakeholder-engaged, patient-centered and community-based participatory research, even these teams may not have sufficiently drawn on expertise across health science disciplines and alternative fields of inquiry. Equity-focused teams are often limited to patients, advocates, community leaders, clinicians and researchers in select disciplines. Their focus is usually limited to a specific disease, population, or approach, with limited ability or impetus to move work into other areas. Often missing are stakeholders who can provide additional insights, approaches and resources, such as funders, entrepreneurs and policy-makers. Stakeholder-engaged team science efforts are also present in relatively few academic and community organizations because there are only a handful of leaders committed, funded, or supported to lead these efforts. Even sites with adequate expertise often have limited infrastructure to efficiently and rapidly respond to emerging problems, and harness expertise outside their current focus. This lack of infrastructure can lead to missed opportunities to innovate, respond to funders and meet institutional priorities. Furthermore, there is a conflict between engagement efforts, known to add significant additional time to research initiatives, and a growing impatience with the years, even decades between research initiation and measurable, long-term impact. 

The development of flexible, readied catalytic frameworks designed to foster communication across scientific and non-scientific divides and promote shared vision can potentiate timely responsiveness to pressing questions, resulting in the more rapid generation of novel solutions to address and ultimately eliminate disparities. If done carefully, these should allay concerns that expanding the types of stakeholders at the table will dilute or silence the voice of underrepresented or vulnerable populations who are just gaining the traction and expertise to shape research agendas and approaches. This process should meet the growing mandate from funders who expect evidence of robust engagement for funding, and from stakeholder communities who are no longer interested in sharing their ideas or allowing access to clients or patients for recruitment, just to be used by academics without a bona fide stakeholder role. 

The accelerator model builds upon a framework of team science and embraces the principles inherent in the notion of building a “community of practice” [6]. Leveraging the skills of cross-disciplinary engagement and collaboration, these vehicles for entrepreneurship allow for participants to share vision and purpose, and pursue novel endeavors with aligned expectations. The transdisciplinary accelerators we describe may optimize our capacity to harness resources and ideas, and to deploy an organized “rapid response” team capable of responding to proposals (“research disaster preparedness”), emerging threats (i.e., lead in water supplies), and opportunities (i.e., harnessing new technologies and genomics discoveries). We hypothesize that these accelerators will spark innovation around specific topics, challenges and populations, generate novel ideas, questions, and approaches, increase stakeholder engagement, introduce new partners to translational research, maximize research quality and efficiency, optimize data democratization and dissemination among target populations, and identify and address root causes of disparities.

## 2. Materials and Methods

As part of the Clinical Translational Science Award (CTSA) from the National Institutes of Health, we created the Community-Academic Research Partnership Board. This group of clinicians, researchers, patients and community advocates was a nidus for collaborative discussions and activities to translate discoveries for diverse communities and generate new ideas for research. The action-oriented board built research collaborations and initiatives, and formed subcommittees focused on specific topics to integrate equity, engagement, team science, and improving health (with a focus on social determinants of health) into the agendas and operations of all groups with whom we interface. We maintain infrastructure to connect stakeholders who represent diverse groups, create a culture of openness, acceptance, respect, generosity and curiosity to encourage sharing of ideas from within and from outside the board, build capacity stakeholders, and maintain transparency in decision making and operations. 

**Figure 1:**
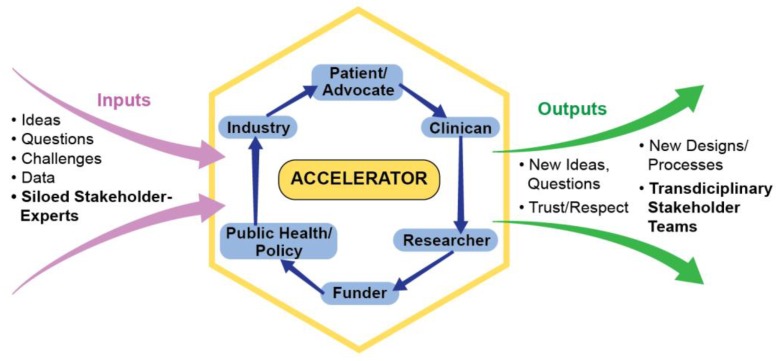
Accelerator model powered by team science.

Upon renewal of the award, with the recommendation of the funder, the board agreed to expand into the Translational Science Board in order to foster communication, collaboration, and transformative translational research across public, private and academic sectors. Board members worked with new private sector partners to determine how best to expand, and decided to revamp the subcommittees to form teams who would endure and innovate beyond one project. As shown in Figure 1, in these “accelerators”, stakeholders collaborate to translate ideas, questions, challenges and data from siloed stakeholders into new ideas, questions, designs and processes posed by a transdisciplinary team with mutual trust and respect. As engines that drive stakeholder-engaged, transdisciplinary team science, accelerators: (1) create teams of experts who are able to draw on each other’s areas of expertise; (2) facilitate experts moving across traditional areas of work into new areas of collaborative research and exploration; (3) foster team science that better addresses community concerns; (4) promote stakeholders sharing their own perspectives and learning to think about research from others’ perspectives; (5) are ready to rapidly generate or respond to new ideas and challenges, funding opportunities and requests from clinical and lay organizations; (6) translate findings for policy, system and environmental changes; and (7) speed up, rather than retard the process of research. 

## 3. Results

Our model presents an innovative way to include stakeholders within each accelerator. Because our board has retained foci on equity and social determinants, an open door to new ideas and sustained efforts to build capacity of all partners, it holds the promise of sparking more effective efforts to reduce racial and ethnic disparities in health. For our results, we describe our formation and projects under development within each accelerator, and explore opportunities for future growth.

### 3.1. Translational Science Board

**Figure 2:**
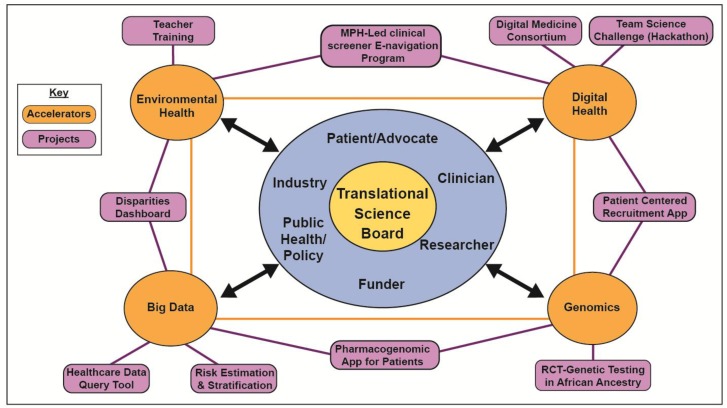
Overview of the Translational Science Board and accelerators.

The new board includes members from the original board and from new sectors and accelerator leaders, as shown in Figure 2.

The accelerators spring from the board. By having funders at the table, the model can influence funding direction and avoid a common peril that even the best ideas are abandoned if not funded after a couple of grant submissions. Private sector stakeholders can maximize support for accelerator products, and introduce new partners to translational research. We began by transforming four groups/activities into accelerators: a cluster of small board, community, and institutional mHealth projects became the Digital Health accelerator; a request from data scientists to inform their expanding field became the Big Data accelerator; a board subcommittee became the Genomics accelerator; and a group who came together to respond to a specific funding opportunity became the Environmental Health accelerator. Accelerator work is summarized in Table 1 and key activities are highlighted below.

### 3.2. Digital Health Accelerator

Mobile applications (apps) are becoming useful for various health needs, integrated with telemedicine and wearables to support fitness, health education, symptom tracking, e-research visits and research data tracking. However, the field is quite fragmented, with over 165,000 apps in healthcare alone. This accelerator aims to bring together numerous small board, community and institutional mHealth projects under one umbrella and change the current situation, where mHealth projects are too rarely developed for or with low-income or multicultural populations [7]. The accelerator-led activities summarized in Table 1 include clinicians, patients, investors, entrepreneurs, researchers, public health leaders and industry, and active collaboration occurs with local, regional and national groups. 

The Digital Medicine Consortium’s Network of Digital Evidence (NODE) Health aims to be an academic home for evidence in digital medicine through a think-tank consortium of academic medical centers and health systems and by bringing together diverse patients, researchers, clinicians, and regulatory bodies. Combining startup spirit with rigor of evidence based medicine, NODE plays a critical role in rapid adoption and evaluation of digital medicine pilots, preventing duplication, standardizing policies and ensuring that diverse patients are part of teams who review apps in order to appropriately engage interests and meet the needs of priority populations [8].

Our board recognized that the majority of trials suffer from delayed or incomplete recruitment, and underrepresentation of minority and low-income individuals [9]. Board clinicians requested that researchers identify a patient-centric method for clinicians (particularly outside academic health centers who feel they and their patients are often the last to know about new discoveries) to disseminate information about ongoing studies to make it easier for people from groups underrepresented in research to learn about research in user-friendly, culturally appropriate, and low-literacy formats and to enroll in trials. Accelerator leaders developed Team4 Cure, an app with a Spanish language module that allows patients to learn about open clinical studies using experiential multimedia (including pictures, videos, texts) from homes, waiting rooms, social media or Google Search, and receive verbal consent forms. The IRB-approved app is also creating videos and a research education module to explain why it could be beneficial for diverse populations to take part in research. This is led by patients and advocates, and testimonials from research veterans from underserved communities are provided, making the platform available across multiple health systems and inserting patient-reported outcomes from the app into electronic health records.

### 3.3. Genomics Accelerator

The impact of genomic medicine is prominent in medical diagnosis, treatment, and risk communication. Stakeholders who may be unfamiliar and uncomfortable with genomics, particularly in terms of any relationship it may have to ethnicity, race or ancestry, are finding themselves in unchartered territory. It is imperative to convene individuals around genetics research that benefits diverse groups [10]. Based on a keen interest in genomic medicine among board patients and advocates, the board created a genomics accelerator comprised of clinicians, patients, researchers, industry and advocates. Current work includes an NIH-funded clinical trial, and development of a pharmacogenomics app to lower the barriers for an individual to find out how their genetic makeup may affect how they respond to certain medications. There is a particular focus on ensuring the app is informative, actionable and will meet the needs of individuals who have little genetic background or have limited health literacy. 

The NIH-funded trial was conceived when a genomics researcher asked to present to the board and explained that while people of African ancestry have increased risk of kidney failure due to numerous socioeconomic, environmental, and clinical factors, variants in the APOL1 gene account for much of the racial disparity associated with hypertensive kidney failure [11]. He asked if the board was interested in determining the impact of translating this knowledge into clinical practice and the board decided it was important to have advocates, patients and clinicians work with researchers to ensure the research would be done respectfully, carefully and capture how patients and clinicians react to testing and return of results. They formed a genomics subcommittee, which became a genomics accelerator. With NIH funding, its members worked to conduct a clinical trial [12], remaining actively involved in every step of the study, and sharing lessons learned with Precision Medicine, NIH leaders, and the White House [13]. They are working with the Digital Health and Big Data accelerators, using the Team4Cure app to recruit patients, and are part of NIH’s IGNITE translational genomics network which helps to engage providers and patients more broadly in genomics-disparities research [14]. They are now including data on social determinants in new studies to determine the interrelationship between environmental, social, clinical and genetic factors on health disparities. 

### 3.4. Big Data Accelerator 

The explosion of research using Big Data has not adequately included a focus on disparities. There are few stakeholders who understand how to obtain and use Big Data, who inform what questions should be asked, and how data discoveries will be used. This accelerator aims to make Big Data more broadly accessible and actionable for a variety of stakeholders. Its members (clinicians, patients, data scientists, disparities researchers, engineers, industry) are developing clinical informatics and translational bioinformatics applications to fuel the democratization of big data in healthcare and community settings [14]. Data scientists are working with stakeholders to provide cutting edge tools and systems to ask data-driven and hypothesis-driven questions using unique toolsets that can handle high volume, velocity, veracity and a variety of data streams. This accelerator has several active projects underway. 

To democratize access to Big Data, disrupt the siloed way data is traditionally stored and used, and move beyond business-as-usual (in which the few uber-users of these data are often divorced from researchers, advocates, clinicians and patients who could expand the questions asked and the actions taken in response to findings), clinicians sparked the development of “HealthBase”. This data query, retrieval and visualization tool allows stakeholders to deploy machine learning algorithms and application programming interfaces for secure and fast access to data at an individual, cohort, or system-wide level. The first query tool developed investigated diabetes across self-reported people of European, African and Latino ancestry to find ancestry-specific disease trajectories and aid in developing personalized therapies [15,16]. 

To make data more actionable, the accelerator is building teams and skillsets of the teams to use data, with diverse stakeholders at the table to facilitate this process. These range from patients who think out of the box, to funders who can add these to their portfolios. The accelerator includes experts in communication and public policy to make use of the findings. To address the shortage of individuals skilled in these areas, the team is developing a comprehensive data science boot camp. One product is a real-time risk estimation and stratification tool to improve outcomes of patients, first among those at risk of unexpected escalation of care during hospitalizations. The team will also provide a risk portal that provides options for shared decision-making, visual analytics and report generation to improve quality of care and reduce disparities. They are working with the digital health accelerator to link EHRs to patient-generated passive data feeds through devices and wearables, which provide an unprecedented amount of data that could unlock information about influences and indicators of health, illness and disparities [17,18].

Clinicians and patients on the board also asked to build a disparities dashboard with accurate data on race and ethnicity that captures key social determinants in EHRs [19]. With enthusiastic support from the system’s Quality Leadership Council, the team conducted pilot interviews with clinic registrars and managers, revealing important challenges in collecting accurate race and ethnicity data. Next, the team developed training materials to enhance data collection, and a framework to implement the dashboard and use data collected to inform interventions to achieve health equity. The dashboard will also include social determinants by linking EHRs and secondary databases with data on neighborhood characteristics to identify new risks for disease and communities that merit increased focus in population health improvement initiatives. 

### 3.5. Environmental Health (EH) Accelerator

One quarter of deaths worldwide are a result of living or working in an unhealthy environment [20]. In areas like NYC, low income and non-White populations are disproportionately exposed to and impacted by environmental risks [21]. The EH accelerator arose when board members formed a transdisciplinary team to respond to a request for proposals to study air quality using citizen science. The group of researchers, clinicians, advocates, device-makers, app-developers, programmers, environmental lawyers, designers and policymakers, who had never met in such a team, came up with very promising, innovative ideas, but could not respond rapidly enough to meet proposal deadlines. Not wanting to disband and lose the team potential they had discovered, they successfully responded to a request to become the Community Outreach and Engagement Core for an NIH-funded center to enhance our understanding of how exposures influence health, development, and risk of disease across the life span. By placing the work of the accelerator in the context of the center, the health system, and national networks of CTSAs and environmental health research centers, it is more effective in promoting science that broadly considers environmental and social risk factors in ongoing research for a wide array of health conditions. 

As shown in Table 1, this accelerator is able to rapidly respond to time-sensitive opportunities such as coordinating health messaging and education around lead in school drinking water, and to create and disseminate culturally and educationally appropriate fact sheets, infographics, videos, and other educational materials and resources for our target urban minority populations. The clinical E-Screener, for example, brings together organizations, city agencies, faculty and staff to connect patients with resources based in an EHR-based environmental health screening tool. Allied health professionals including social workers, community health workers and masters of public health (MPH) students will help physicians and patients with the screening, address positive results and connect them with resources to address concerns. An e-screener app will serve as a bilingual, low-literacy, user-friendly interface for patients to select resources that meet their needs (i.e., healthy housing interventions and food pantries). Once validated, this practice-based model can expand across health systems and communities, and serve as a platform to launch additional experiential and service learning for masters trainees in other disciplines such as social work, education and bioinformatics.

The accelerator also supports professional development course for teachers. In “Citizen Science and Social Justice in Your Neighborhood,” led by the Children’s Environmental Literacy Foundation, teachers from low-income and multicultural schools receive professional credits for attending a course that packages the latest in environmental health science into a digestible format for students. Investigators co-lead the course and contribute to lesson plans that teachers then implement in their classrooms, thereby inspiring researchers to identify consumer-friendly implications of their work and transmit them in a user-friendly, actionable fashion. A citizen scientist component is structured into the curriculum so students can learn how to collect, analyze and act on data with accessible tools such as smart phones. The program model by which professional credits are offered to promote incorporation of emerging science into education is a model that could be broadly replicated with other multi-disciplinary teams such as reproductive health. 

## 4. Discussion

Initially hesitant, our transdisciplinary team expanded a more traditional board of community and clinical stakeholders to an engagement board including members of the private sector, while taking care not to dilute community voice and with careful attention to the principles of CBPR, patient-centered research, and team science. Unable to rapidly respond to institutional requests and funder opportunities, we responded to increasing concerns about research teams working in silos by forming accelerators that foster the development and implementation of research that can impact health. By organizing the topic-specific accelerators with broad, diverse sets of stakeholders working at the intersection of real world problems and emerging methods, opportunities and technologies, this model has the potential to transform research. 

Several aspects of this work deserve specific mention. Firstly, by collaborating with various groups (patients, clinicians, researchers, public health officials, funders, industry) we create the right atmosphere for team science. We are able to find the right collaborators and link them to one of their interests, even if it is outside of their comfort zone, and help them work with people they would not normally meet, let alone collaborate with. The work leads to fertilization across disciplines. This includes prioritizing patients, advocates and community clinicians as equally valued team members who proactively, not just reactively, contribute ideas and strategies that will benefit the communities unjustly impacted by disparities in healthcare and health. It has become more routine for teams to critically evaluate whether the right people are at the table both across the health system and in the larger community, whether it is a clinician, a community partner, investigator with unique expertise or another expert. 

Secondly, we can expand the innovative products of accelerators, because members form networks of networks that reach their respective stakeholder groups. The program to provide environmental education to teachers will extend to other types of science education and expand to include additional disciplines. Research on race, genomics and kidney disease will be extended to obesity. Big data in hospitals will be supplemented with social determinants data. 

Focusing collective expertise for collaborations to improve our understanding of illness improves health, makes research more relevant, feasible, sustainable and scalable, and fosters the translation of research into action at the individual, community and societal levels. Substantive, productive engagement requires: (1) contribution of key stakeholders in all phases of research; (2) development of trust, collaboration, shared decision-making and shared ownership of research; (3) assurance that findings and knowledge benefit all partners; (4) adoption of a bi-directional, co-learning process that recognizes and embraces skills, resources and assets of all stakeholders; (5) commitment to long-term research relationships; and (6) emphasis on capacity building and sustainability [1]. We aim to foster innovation in research by reaching out broadly and bringing together diverse partners to think through complex health issues and to meaningfully address them. 

There are some challenges to this work. Building teams takes time, skill and regular, effective communication with partners to elicit and address their concerns. It is important to ensure all partners are on the same page to help direct partners to work toward common goals, approach or propose new research questions, disseminate research findings or inform public policy. It may be difficult for researchers to cede control and allow others to help them develop new questions and strategies. It may be difficult for patients, clinicians and community partners to trust that outsiders will respect and not take advantage of their ideas and experiences. It may be difficult for entrepreneurs to operate with unfamiliar partners. However, it is precisely this mix of individuals that can truly innovate. Despite challenges, accelerators and the network of networks they represent could be very beneficial in moving from isolated research projects to implemented, sustained and scaled solutions. 

## 5. Conclusions

Team science is at the core of the accelerator model, which has the power to maximize research quality and efficiency, improve patient care and stakeholder engagement, and optimize data democratization and dissemination among target populations. The infrastructure connects a network of diverse stakeholders, research participants, and innovative team science programs in accelerators to spark innovation around specific topics, challenges and populations. These accelerators generate novel ideas, increase stakeholder engagement, introduce new partners to translational research within and outside of their institutions, and bring in funders and private sector stakeholders to maximize innovation and support for accelerator products. This innovative model has led our teams to ask new questions, entice new funders, develop new methods to recruit diverse patients, build and utilize big data, employ new research methods, and disseminate lessons learned to inform healthcare systems, policies and communities.

## Figures and Tables

**Table 1:** Accelerator activities, community input, and outcomes. NODE: Network of Digital Evidence.

Accelerator	Projects	Community Input	Outcomes
**Digital Health**: Engaging diverse stakeholders in expansion of mHealth to meet the needs of diverse patients, clinicians and communities.	“NODE” advance evidence base, adoption, scaling of apps	New review criteria which is relevant and usable for diverse populations, and added community reviewers.	More diverse apps and inclusion of community in mHealth processes.
	“Team4Cure”—pt-centered e-recruitment to increase diversity in clinical trials	Spanish version provided and tailored for diverse populations. Research 101 and research hero videos provided, as well as added verbal consent info.	Increased recruitment of diverse people, increased recruitment in community practices.
	“App Chat” building mHealth capacity among diverse stakeholders	The idea was generated by community partners.	Increased community capacity and relevance.
	Team Science Hackathons	Made challenges focused on diverse populations, added community judging included.	New mHealth tools, increased community capacity.
**Genomics**: Diversifying clinical and community input into translational genomics	Building of translational diversity and a disparities research operation, a hub for developing new ideas and grants	Community co-led all aspects of RCT, uncovered challenges, and disseminated lessons nationally/internationally. The community published the manuscript.	Met recruitment goals, two new grants awarded, two submitted with new partners.
	Pharmacogenomics app	Information made useful/accessible to diverse populations.	New diversity focus by entrepreneur.
**Big Data**: Making data more accessible and actionable for diverse research, clinical, patient communities	Big data query tool: More accessible data	Diverse patients, clinicians helped to pose new questions and ways to build query for more democratic access to data.	Found ancestry-specific disease trajectories, building therapeutics.
	Risk stratification tool: More actionable data	Front line clinicians helped build a tool for them to identify at-risk patients and use shared decisions for better care and reduction of disparities.	Early warning system for inpatients who will need more care.
	Disparities dashboard: to identify and address system-wide disparities	Proposed dashboard, training for data collection, and categories to be collected, addition of social determinants.	Data for >7000 clinicians, >4 million patient visit for evaluations and interventions.
**Environmental Health** (EH): Expanding EH research focus on diverse communities	Research dissemination to priority communities/ clinicians for local benefit	Developed and implemented strategies for low literacy, multilingual dissemination, training held to translate findings to actions.	Coordinated messages with respect to lead, pesticides, smoking, and plastics.
	Clinical screening e-navigator: EHR screening and linking of patients to local resources	Co-developed screening questions, identified local resources for linkage.	One grant obtained, integrated into the health system.
	Professional development course for inner city teachers to teach EH	Pitched the idea, identified teachers and structure for the course and built citizen science component.	Teachers trained, planned expansion.
	Formation of a community outreach-engagement core for researchers	Became go-to group for stakeholders to develop EH grants and activities focused on diversity.	Became core for new center, grant awarded.
